# Supplementary material for: Resolving the full spectrum of human genome variation using Linked-Reads
Source: Genome Res. 2019 Apr;29(4):635–45. doi: 10.1101/gr.234443.118 (PMC6442396; doi:10.1101/gr.234443.118)
Supplement: Supplemental Material [file supp_gr.234443.118_Supplemental_longranger-2.2.zip › longranger-2.2.tar.bz2!/lr2.2-public/lib/stan/cmdstan/stan/doc/api/html/diag__e__static__hmc_8hpp_source.html]

Stan: src/stan/mcmc/hmc/static/diag\_e\_static\_hmc.hpp Source File


|  |  |
| --- | --- |
|  | Stan  2.5.0  probability, sampling & optimization |


- Main Page
- Related Pages
- Namespaces
- Classes
- Files

- File List
- File Members

All Classes Namespaces Files Functions Variables Typedefs Enumerations Enumerator Friends Macros Pages

- src
- stan
- mcmc
- hmc
- static

diag\_e\_static\_hmc.hpp

Go to the documentation of this file.

1 #ifndef STAN\_\_MCMC\_\_DIAG\_\_E\_\_STATIC\_\_HMC\_\_BETA

2 #define STAN\_\_MCMC\_\_DIAG\_\_E\_\_STATIC\_\_HMC\_\_BETA

3

4 #include <stan/mcmc/hmc/static/base\_static\_hmc.hpp>

5 #include <stan/mcmc/hmc/hamiltonians/diag\_e\_point.hpp>

6 #include <stan/mcmc/hmc/hamiltonians/diag\_e\_metric.hpp>

7 #include <stan/mcmc/hmc/integrators/expl\_leapfrog.hpp>

8

9 namespace stan {

10

11  namespace mcmc {

12

13  // Hamiltonian Monte Carlo on a

14  // Euclidean manifold with diagonal metric

15  // and static integration time

16

17  template <typename M, class BaseRNG>

18  class diag\_e\_static\_hmc: public base\_static\_hmc<M,

19  diag\_e\_point,

20  diag\_e\_metric,

21  expl\_leapfrog,

22  BaseRNG> {

23

24  public:

25

26  diag\_e\_static\_hmc(M &m, BaseRNG& rng, std::ostream\* o = &std::cout, std::ostream\* e = 0):

27  base\_static\_hmc<M, diag\_e\_point, diag\_e\_metric, expl\_leapfrog, BaseRNG>(m, rng, o, e)

28  { this->name\_ = "Static HMC with a diagonal Euclidean metric"; }

29

30

31  };

32

33  } // mcmc

34

35 } // stan

36

37

38 #endif

stan::mcmc::base\_mcmc::name\_

std::string name\_

**Definition:** base\_mcmc.hpp:38

base\_static\_hmc.hpp

stan::mcmc::diag\_e\_static\_hmc::diag\_e\_static\_hmc

diag\_e\_static\_hmc(M &m, BaseRNG &rng, std::ostream \*o=&std::cout, std::ostream \*e=0)

**Definition:** diag\_e\_static\_hmc.hpp:26

stan::mcmc::base\_static\_hmc

**Definition:** base\_static\_hmc.hpp:18

stan::mcmc::expl\_leapfrog

**Definition:** expl\_leapfrog.hpp:12

expl\_leapfrog.hpp

diag\_e\_metric.hpp

stan::math::e

double e()

Return the base of the natural logarithm.

**Definition:** constants.hpp:86

stan::mcmc::diag\_e\_point

**Definition:** diag\_e\_point.hpp:12

diag\_e\_point.hpp

stan::mcmc::diag\_e\_static\_hmc

**Definition:** diag\_e\_static\_hmc.hpp:18

stan::mcmc::diag\_e\_metric

**Definition:** diag\_e\_metric.hpp:16


---

[ Stan Home Page ]

*© 2011–2014,
Stan Development Team.*
